# Supplementary material for: Extending carbon chemistry at high-pressure by synthesis of CaC2 and Ca3C7 with deprotonated polyacene- and para-poly(indenoindene)-like nanoribbons
Source: Nat Commun. 2024 Apr 2;15:2855. doi: 10.1038/s41467-024-47138-2 (PMC10987516; doi:10.1038/s41467-024-47138-2)
Supplement: Supplementary file 1 — Supplementary Information [file 41467_2024_47138_MOESM1_ESM.pdf]

Extending carbon chemistry at high-pressure by synthesis of CaC_2 and Ca_3C_7 with deprotonated polyacene- and *para*-poly(indenoindene)-like nanoribbons

Saiana Khandarkhaeva[1,2], Timofey Fedotenko[3], Alena Aslandukova[1], Fariia Iasmin Akbar[1], Maxim Bykov[4], Dominique Laniel[2,5], Andrey Aslandukov[2], Uwe Ruschewitz[4], Christian Tobeck[4], Björn Winkler[6], Stella Chariton[7], Vitali Prakapenka[7], Konstantin Glazyrin[3], Carlotta Giacobbe[8], Eleanor Lawrence Bright[8], Maxim Belov[9], Natalia Dubrovinskaia[2,9] and Leonid Dubrovinsky[1]*

[1]Bayerisches Geoinstitut, University of Bayreuth, Universitätstraβe 30, 95440 Bayreuth, Germany

[2]Material Physics and Technology at Extreme Conditions, Laboratory of Crystallography University of Bayreuth, Universitätstraβe 30, 95440 Bayreuth, Germany

[3]Deutsches Elektronen-Synchrotron DESY, Notkestraße. 85, 22607 Hamburg, Germany

[4]Institute of Inorganic Chemistry, University of Cologne, Greinstraße 6, 50939 Cologne, Germany

[5]Centre for Science at Extreme Conditions and School of Physics and Astronomy, University of Edinburgh, Edinburgh, UK

[6]Institute of Geosciences, Goethe University Frankfurt, Altenhöferallee 1, 60438 Frankfurt, Germany

[7]Center for Advanced Radiation Sources, The University of Chicago, 5640 S. Ellis, 60637 Chicago, Illinois, USA

[8]European Synchrotron Radiation Facility, CS 40220, 38043 Grenoble Cedex 9, France

[9]Department of Physics, Chemistry and Biology (IFM), Linköping University, SE-581 83 Linköping, Sweden

*Corresponding author: leonid.dubrovinsky@uni-bayreuth.de

Supplementary Information

Supplementary figures.

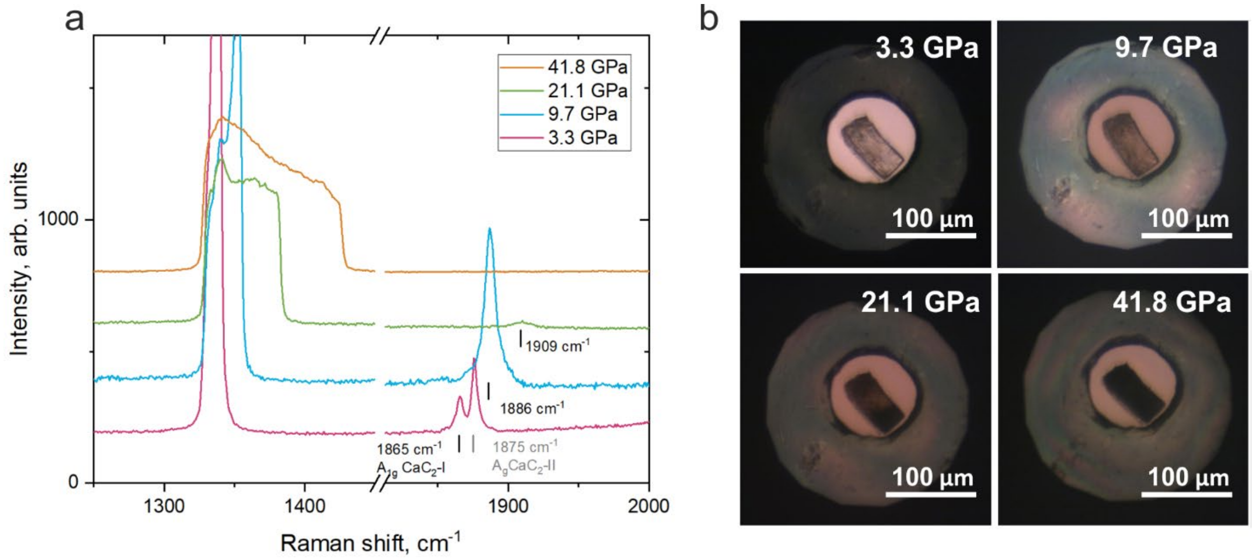

Supplementary Figure 1. Raman spectra (a) and microphotographs (b) of a CaC_2 sample taken upon its compression at room temperature in DAC #1. Raman spectra (a) were recorded on a DilorXY system equipped with an Ar (excitation wavelength: 514 nm) laser source. Ticks highlight the vibrational modes of two polymorphs: tetragonal CaC_2-I (black) and monoclinic CaC_2-II (grey). The spectrum recorded at 3.3 GPa from the sample in DAC #1 reveals the vibrational modes which can be assigned to the two CaC_2 polymorphs, tetragonal CaC_2-I[1,2] and monoclinic CaC_2-II[1,2]. At ambient conditions, the A_{1g} mode of CaC_2-I and the A_g and B_g modes of CaC_2-II are found at 1859 cm^{-1}, 1871 cm^{-1}, and 1874 cm^{-1}, respectively. The Raman modes we observed at 3.3 GPa (1865 cm^{-1} and 1875 cm^{-1}) are slightly shifted to the higher frequencies, as expected to happen under compression. Above ~5 GPa, CaC_2-II is known to transform into CaC_2-I, thus, the vibrational mode of CaC_2-II should not be present in the spectrum above this pressure. This is in accordance with our observation, as the Raman spectrum at 9.7 GPa features a single peak. At ~25 GPa the Raman spectrum becomes featureless, suggesting amorphization of CaC_2.[1] As CaC_2-II was not detected by XRD in preselected crystals, we assume that only a minor amount of this phase was present in the sample loaded into the DAC. Microphotographs (b) reveal changes in the visual appearance of the sample with increasing pressure: the initially transparent crystal becomes translucent at ~10 GPa and then opaque above ~25 GPa.

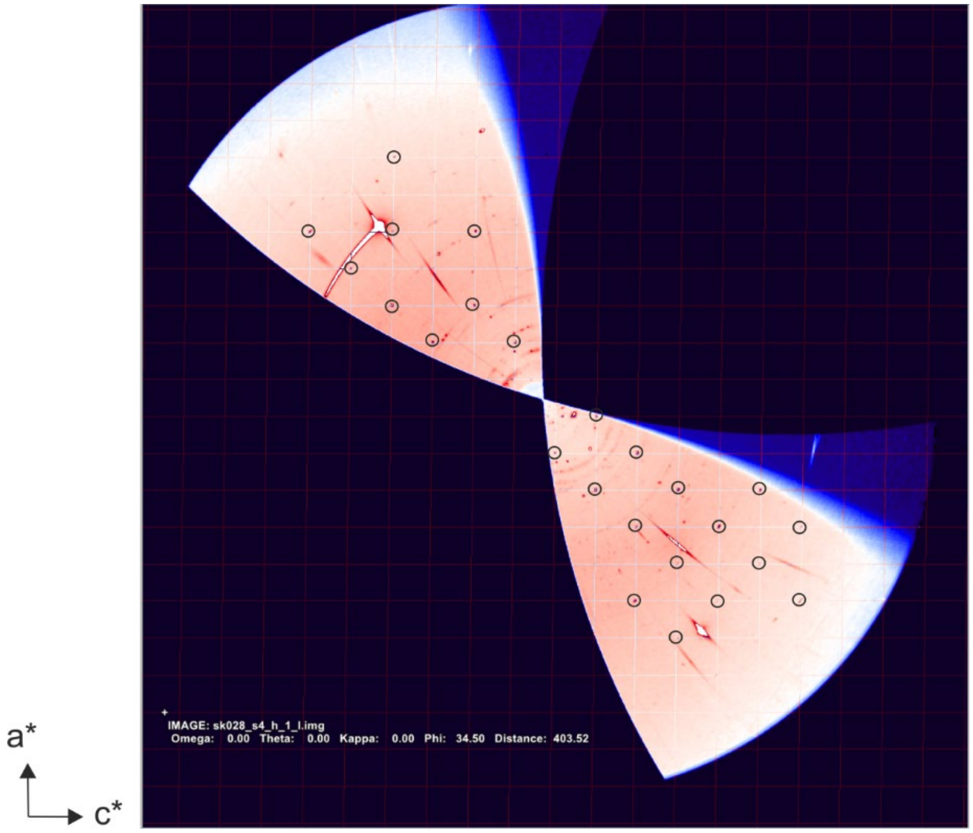

Supplementary Figure 2. Reciprocal space reconstruction of the (*h1l*) plane of the single-crystal domain of the high-pressure CaC_2 polymorph (HP-CaC_2, space group *Immm*) observed after laser heating CaC_2 at 2400(100) K at 44(1) GPa. Spots in the black circles indicate reflections which belong to a single grain of the best quality.

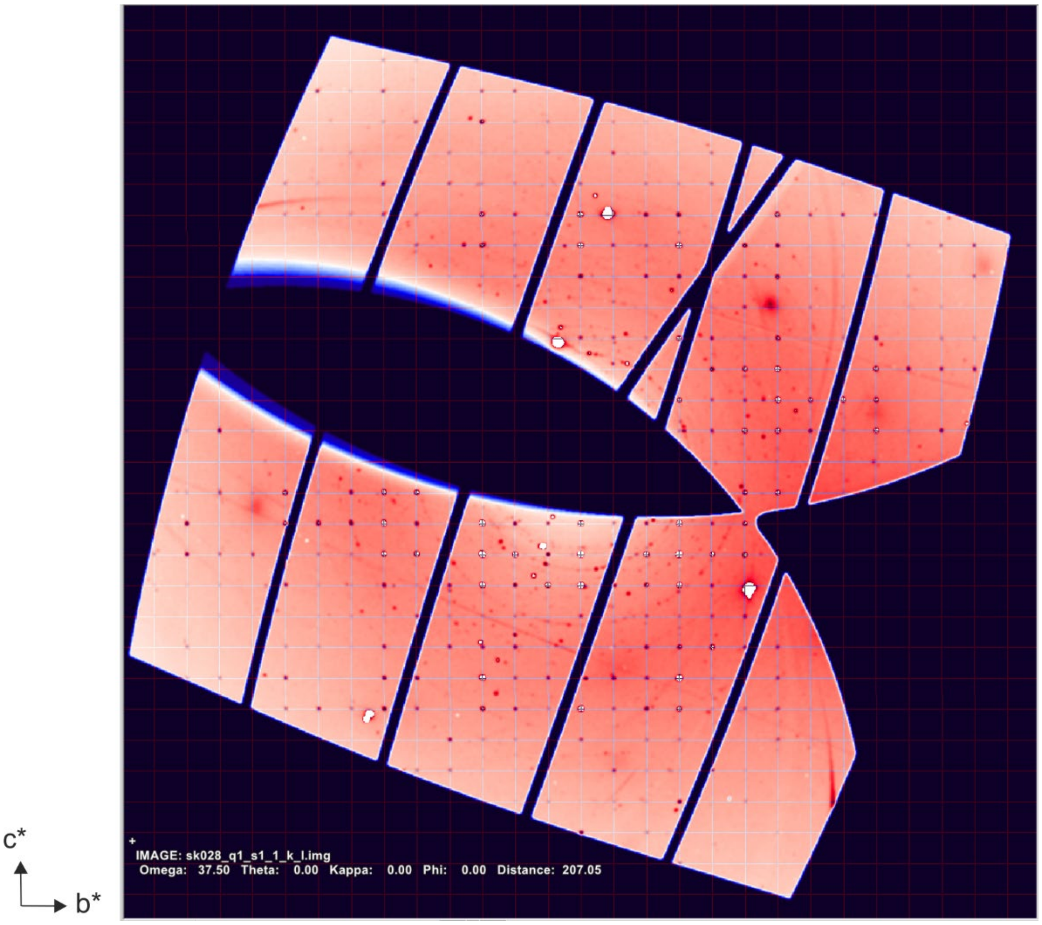

Supplementary Figure 3. Reciprocal space reconstruction of the (*1kl*) single-crystal domain of the Ca_3C_7 phase observed after laser-heating CaC_2 at 2350(150) K at 38(1) GPa. Diffraction peaks, which belong to the single-crystalline grain of the best quality used for structure solution and refinement, are seen at the nodes of the reciprocal lattice.

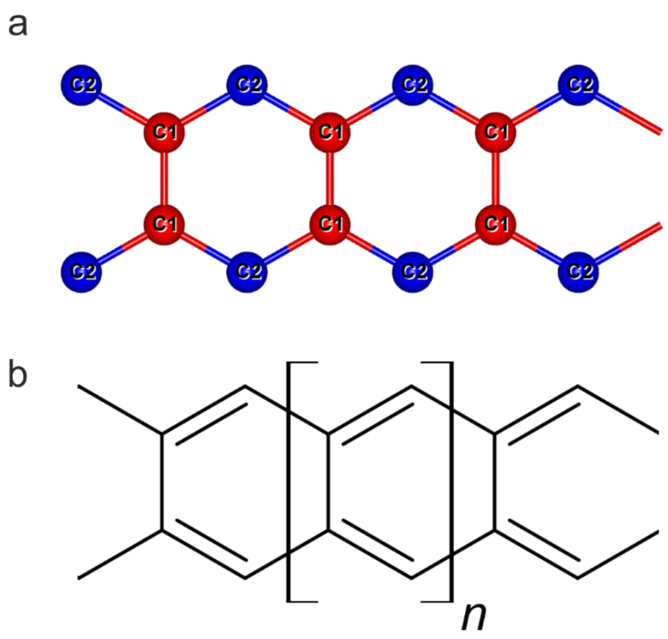

Supplementary Figure 4. A comparison of a carbon poly-anion in the crystal structure of HP-CaC_2 (a) and polyacene (b).

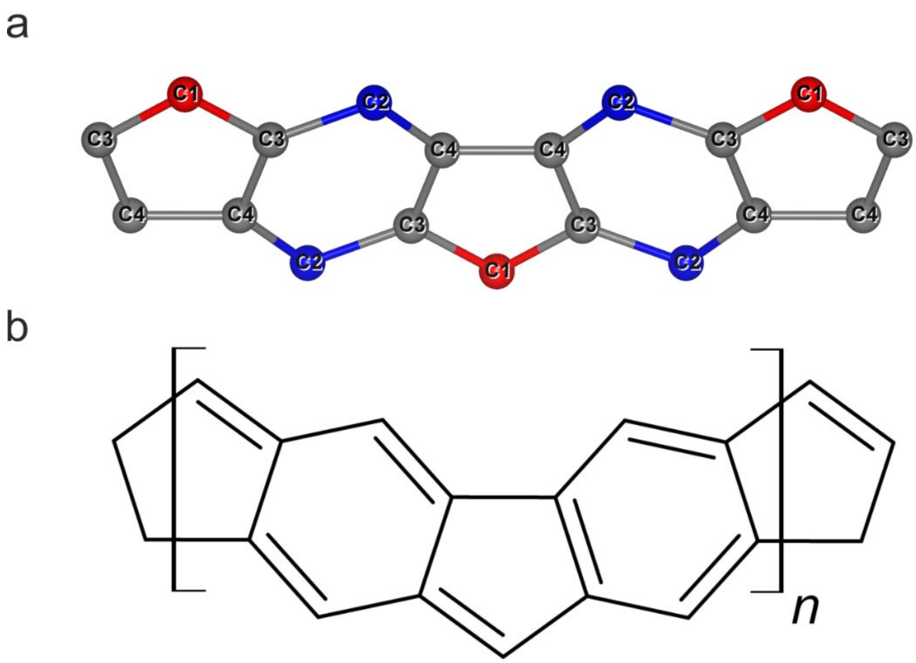

Supplementary Figure 5. A comparison of a carbon poly-anion in the crystal structure of Ca_3C_7 (a) and *para*-type poly(indenoindene) (b).

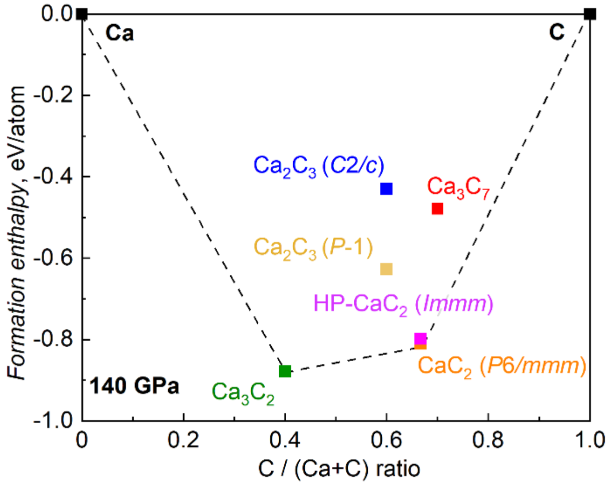

Supplementary Figure 6. The calculated convex hull in the Ca-C binary system for predicted and observed calcium carbides at 140 GPa. The HP-CaC_2 phase lies on the convex hull (black dashed line) and is thus thermodynamically stable at this pressure.

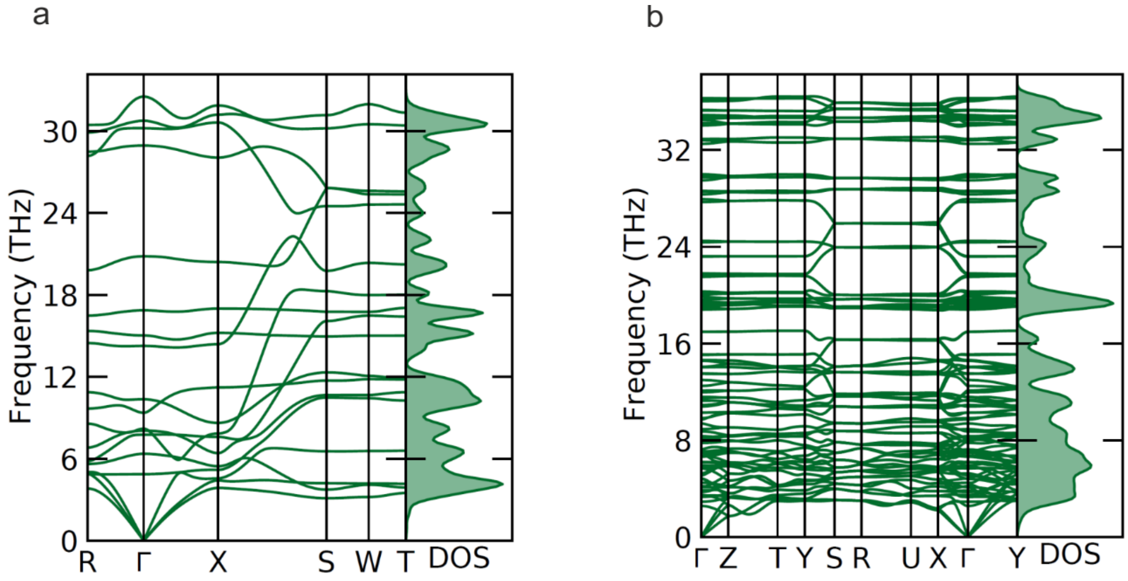

Supplementary Figure 7. Calculated phonon dispersion curves of HP-CaC_2 (a) and Ca_3C_7 (b) at 1 bar.

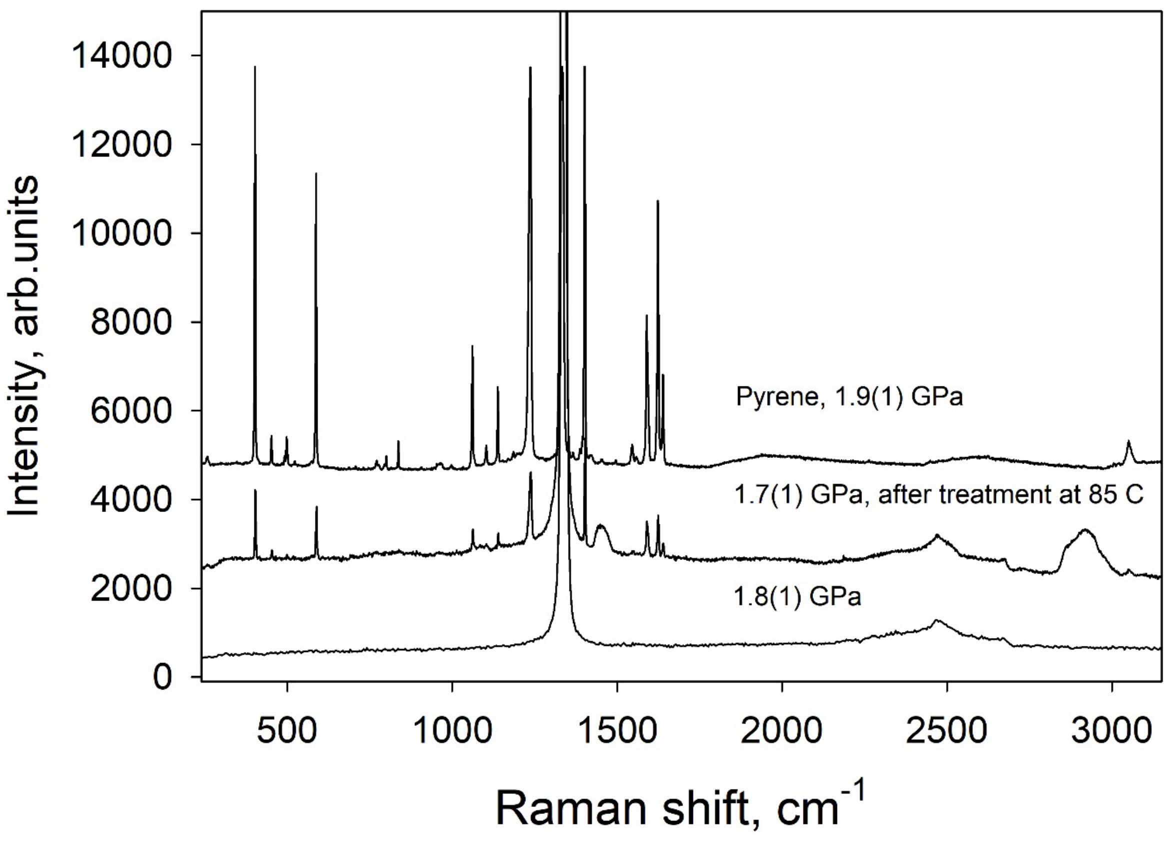

Supplementary Figure 8. Raman spectra recorded after high-pressure high temperature treatment of a mixture of calcium carbide and calcium hydroxide (lower and middle lines), and a spectrum of pyrene ($C_{16}H_{10}$) (upper line). A mixture of CaC_2-I and $Ca(OH)_2$ in the approximate proportion of 1:1 was loaded into a DAC chamber, compressed to ~40 GPa, and the whole sample was double-sided laser-heated between 2500 K and 3000 K. That resulted in a transformation of CaC_2 into high-pressure phases and a decomposition of $Ca(OH)_2$ into CaO (B1-structured phase) and ice. On decompression at ambient temperature, a featureless Raman spectrum was observed down to ~1.8 GPa (lower spectrum). At this pressure, the DAC was warmed at 85°C (above the melting point of water) using a hot plate for 72 hours. Several Raman modes observed belong to C-H vibrations of saturated (2800-3000 cm^{-1}) and aromatic (above 3000 cm^{-1}) hydrocarbons. Moreover, some sample positions (middle spectrum) display Raman spectra that closely resemble those of pyrene ($C_{16}H_{10}$). In order to demonstrate this, pure pyrene was loaded in the DAC and compressed to ~1.9 GPa (upper spectrum).

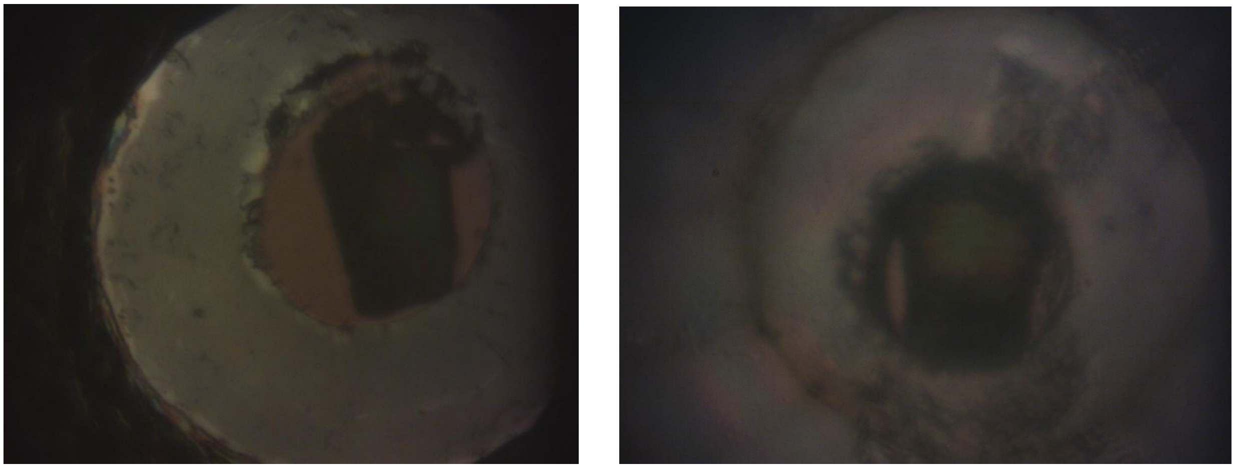

Supplementary Figure 9. Photos of the samples after laser heating at ~38 GPa (left, pressure chamber diameter ~100 µm, Experiment #2_2 in Table S1) and at ~93 GPa (right, pressure chamber diameter ~55 µm, Experiment #6_1 in Table S1). After laser heating the material remains dark.

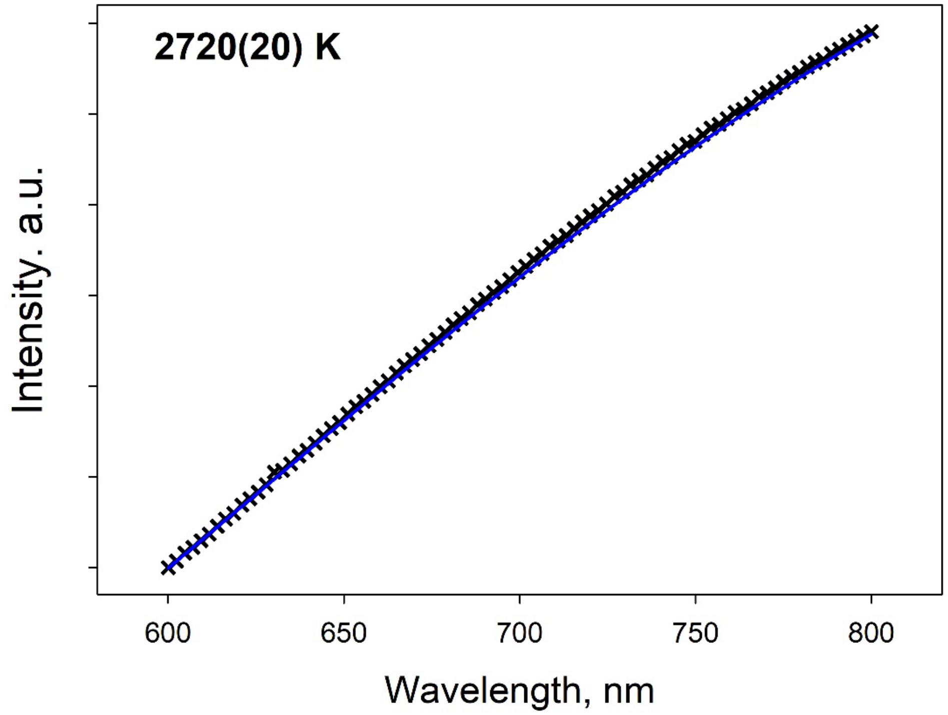

Supplementary Figure 10. Example of fitted with Planck function (blue line) thermal radiation spectra (black crosses) collected upon laser heating of DAC #7.

Supplementary tables

Supplementary Table 1. Summary of experiments conducted in the present study.

Experiment	Phases observed*[3]	*a*, Å	*b*, Å	*c*, Å	V, Å^3	Pressure, GPa	Temperature, K
**#1**	Ca_3C_7	4.715(3)	8.288(4)	8.803(3)	344.0(3)	44(2)	2400(100)
	HP-CaC_2	2.5622(3)	6.024(2)	6.689(2)	103.24(5)		
	B1 CaO	4.444(1)			87.77(5)		
**#2_2***	Ca_3C_7	4.768(4)	8.335(1)	8.856(1)	351.9(3)	38(1)	2350(150)
	B1 CaO	4.4764(4)			89.70(1)		
**#2_3**	Ca_3C_7	4.859(6)	8.385(2)	8.923(2)	363.6 (4)	30(2)**	
	B1 CaO	4.5231(3)			92.54(1)		
**#3**	Ca_3C_7	4.727(5)	8.316(2)	8.791(2)	345.6(3)	44(1)	2300(200)
	B1 CaO	4.4399(4)			87.53(2)		
**#4**	Ca_3C_7	4.720(3)	8.277(4)	8.772(4)	342.7(3)	47(3)	2300(300)
	HP-CaC_2	2.584(1)	6.060(1)	6.706(2)	105.0(5)	47(3)	
**#5**	HP-CaC_2	2.541(1)	5.946(4)	6.598(2)	99.69(8)	52(1)	2400(200)
**#6_1***	HP-CaC_2	2.4965(6)	5.782(3)	6.295(3)	90.87(7)	93(1)	3000(300)
**#6_2***	HP-CaC_2	2.454(2)	5.680(2)	6.141(5)	85.59(10)	139(2)	3300(300)
**#6_3**	HP-CaC_2	2.4667(3)	5.665(2)	6.120(2)	85.53(3)	144(2)	
**#7**	Hydrocarbons*[4]					45(2)	2600(200)-3100(200)

Note: Supplementary Table 1 includes a list of phases observed as single crystal materials in each experiment, their structural parameters, and pressure-temperature conditions of the synthesis. The first digit in the number of an experiment indicates the number of the DAC; the second digit, if given – the number of the experiment conducted in the same DAC.

All the DACs were laser heated in the home laboratory at the BGI. An asterisk (*) in the number indicates that additional laser-heating was done at the extreme condition beamline 13-IDD at the Advanced Photon Source (APS, Chicago, USA). A double asterisk (**) indicates room temperature decompression. All diffraction measurements were made at room temperature on pressurized, but temperature-quenched samples after laser heating.

*[3] DACs #1-#3, and #7: commercial technical grade CaC_2 was used; DACs #4-#6 were loaded with pure pre-synthesized CaC_2.

*[4] Detected by Raman spectroscopy on decompression below 2 GPa.

Supplementary Table 2. Details of the crystal structure refinements and crystallographic data (fractional atomic coordinates, isotropic(*) or equivalent isotropic displacement parameters (Å^2)) for the HP-CaC_2 phase at 44(1) GPa in comparison to the corresponding DFT-relaxed structure.

Chemical formula	CaC_2 (experiment)	CaC_2 (theory)
Pressure, GPa	44(1)	46.2
M_r	64.1	64.1
Crystal system, space group, Z	Orthorhombic, *Immm*, 4	Orthorhombic, *Immm*, 4
Temperature (K)	293	0
a, b, c (Å)	2.5622(3), 6.0244(17), 6.6891(15)	2.56785, 5.99788, 6.69842
V (Å^3)	103.24(5)	103.17
Wyckoff site, fractional atomic coordinates (x, y, z), and U_{iso}*/U_{eq} (Å^2):		
Ca1 4i	0, 0, 0.2039(2); 0.0072(5)	0, 0, 0.20412
C1 4g	0, 0.3824(14), 0; 0.0048(11)*	0, 0.3761, 0
C2 4h	0, 0.2422(16), 0.5; 0.0062(10)*	0, 0.24209, 0.5
C1-C1 distance (Å)	1.417(4)	1.45612
C1-C2 distance (Å)	1.484(5)	1.47397
Radiation type	X-ray, λ = 0.2885 Å	-
Radiation source	P02.2, Petra III, DESY	-
μ (mm^{-1})	0.41	-
Crystal size (mm)	0.002×0.002×0.002	-
T_{min}, T_{max}	0.623, 1	-
No. of measured, independent and observed [$I > 3\sigma(I)$] reflections	291, 134, 96	-
θ_{min}, θ_{max}	3.4°, 15°	-
hkl range	$h = -4 \rightarrow 4$<br>$k = -7 \rightarrow 9$<br>$l = -9 \rightarrow 10$	-
R_{int}	0.105	-
$(\sin \theta/\lambda)_{max}$ (Å^{-1})	1.037	-
$R[F^2 > 2\sigma(F^2)]$, $wR(F^2)$, S	0.076, 0.105, 1.20	-
No. of reflections	122	-
No. of parameters	9	-
$\Delta\rho_{max}$, $\Delta\rho_{min}$ (e Å^{-3})	1.87, −1.75	-

Supplementary Table 3. Details of the crystal structure refinements and crystallographic data (fractional atomic coordinates, isotropic(*) or equivalent isotropic displacement parameters (Å^2)) for the Ca_3C_7 phase at 38(1) GPa in comparison to the corresponding DFT-relaxed structure.

Chemical formula		Ca_3C_7 (experiment)	Ca_3C_7 (theory)
Pressure, GPa		38(1)	39.9
M_r		204.3	204.3
Crystal system, space group, *Z*		Orthorhombic, *Pnma*, 4	Orthorhombic, *Pnma*, 4
Temperature (K)		293	0
a, *b*, *c* (Å)		4.768(4), 8.3350(12), 8.8558(13)	4.7667, 8.3514, 8.8429
V (Å^3)		351.9(3)	352.02
Wyckoff site, fractional atomic coordinates (*x*, *y*, *z*), and U_{iso}*/U_{eq} (Å^2):			
Ca1	4*c*	0.0517(3), 0.25, 0.33976(5); 0.0082(3)	0.0478, 0.25, 0.3414
Ca2	8*d*	0.19561(19), 0.57441(4), 0.35336(4); 0.0081(3)	0.19018, 0.57458, 0.35349
C1	4*c*	0.1913(12), 0.25, 0.6330(2); 0.0070(4)*	0.19494, 0.25, 0.63319
C2	8*d*	0.2158(8), 0.0535(2), 0.1205(2); 0.0085(3)*	0.21199, 0.05162, 0.12039
C3	8*d*	0.1818(8), 0.6127(2), 0.06479(18); 0.0076(3)*	0.18236, 0.61242, 0.065
C4	8*d*	0.0172(8), 0.1627(2), 0.04884(18); 0.0070(3)*	0.01594, 0.16307, 0.05015
Radiation type		X-ray, λ = 0.29521 Å	-
Radiation source		13IDD, GSECARS, APS	-
μ (mm^{-1})		0.39	-
Crystal size (mm)		0.002×0.002×0.002	-
T_{min}, T_{max}		0.547, 1	-
No. of measured, independent and observed [$I > 3\sigma(I)$] reflections		888, 381, 347	-
θ_{min}, θ_{max}		1.9°, 15.0°	-
hkl range		$h = -3 \to 4$<br>$k = -13 \to 13$<br>$l = -13 \to 14$	-
R_{int}		0.016	-
$(\sin \theta/\lambda)_{max}$ (Å^{-1})		0.879	-
$R[F^2 > 2\sigma(F^2)]$, $wR(F^2)$, S		0.027, 0.069, 1.025	-
No. of reflections		381	-

No. of parameters	31	-
$\Delta\rho_{max}$, $\Delta\rho_{min}$ (e Å^{-3})	0.47, −0.52	-

Supplementary Table 4. Results of charge distribution analysis using Bader charge calculations based on the computed charge density from the relaxed structures of HP-CaC_2 and Ca_3C_7 carbides at 40 GPa and 140 GPa.

Phase	Atom and Wyck. site	Bader charges at 40 GPa	Bader charges at 140 GPa
HP-CaC_2	Ca1 / 4*i*	1.191	1.065
	C1 / 4*g*	-0.378	-0.338
	C2 / 4*h*	-0.813	-0.727
Ca_3C_7	Ca1 / 4*c*	1.227	1.096
	Ca2 / 8*d*	1.232	1.109
	C1 / 4*c*	-0.718	-0.631
	C2 / 8*d*	-0.798	-0.693
	C3 / 8*d*	-0.338	-0.307
	C4 / 8*d*	-0.351	-0.342

Supplementary Table 5. Details of the crystal structure refinements and crystallographic data (fractional atomic coordinates, isotropic* or equivalent isotropic displacement parameters (Å^2)) for the HP-CaC_2 phase at 144(2) GPa.

Chemical formula		CaC_2
Pressure, GPa		144(2)
M_r		64.1
Crystal system, space group, *Z*		Orthorhombic, *Immm*, 4
Temperature (K)		293
a, *b*, *c* (Å)		2.4667(15), 5.665(2), 6.61210(13)
V (Å^3)		85.53(19)
Ca1	4*i*	0, 0, 0.2018(2); 0.0069(9)
C1	4*g*	0, 0.3755(12), 0; 0.0036(13)*
C2	4*h*	0, 0.2464(13), 0.5; 0.0068(15)*
C1-C1 distance (Å)		1.410(5)
C1-C2 distance (Å)		1.414(5)
Radiation type		X-ray, λ = 0.2952 Å
Radiation source		ID11, ESRF
μ (mm^{-1})		0.48
Crystal size (mm)		0.0005×0.0005×0.0005
T_{min}, T_{max}		0.624, 1
No. of measured, independent and observed [$I > 3\sigma(I)$] reflections		196, 118, 62

θ_{min}, θ_{max}	2.9°, 19.4°
hkl range	$h = -4 \rightarrow 4$<br>$k = -11 \rightarrow 11$<br>$l = -10 \rightarrow 4$
R_{int}	0.020
$(\sin \theta/\lambda)_{max}$ (Å^{-1})	1.037
$R[F^2 > 2\sigma(F^2)]$, $wR(F^2)$, S	0.044, 0.115, 1.06
No. of reflections	62
No. of parameters	9
$\Delta\rho_{max}$, $\Delta\rho_{min}$ (e Å^{-3})	0.64, −1.05

References.

1. Wang, L.; Huang, X.; Li, D.; Huang, Y.; Bao, K.; Li, F.; Wu, G.; Liu, B.; Cui, T. Pressure-Induced Structural Transformation of CaC2. J. Chem. Phys. 2016, 144, 194506. https://doi.org/10.1063/1.4948705.
2. Konar, S.; Nylén, J.; Svensson, G.; Bernin, D.; Edén, M.; Ruschewitz, U.; Häussermann, U. The Many Phases of CaC_2. J. Solid State Chem. 2016, 239, 204–213. https://doi.org/10.1016/j.jssc.2016.04.030.
